# Uterine Artery Embolization Combined with Subsequent Suction Evacuation as Low-Risk Treatment for Cesarean Scar Pregnancy

**DOI:** 10.3390/diagnostics11122350

**Published:** 2021-12-14

**Authors:** Roxana Bohiltea, Ionita Ducu, Bianca Mihai, Ana-Maria Iordache, Bogdan Dorobat, Emilia Maria Vladareanu, Stefan-Marian Iordache, Alexia-Teodora Bohiltea, Nicolae Bacalbasa, Cristiana Eugenia Ana Grigorescu, Valentin Varlas

**Affiliations:** 1 Discipline of Obstetrics and Gynecology, “Carol Davila” University of Medicine and Pharmacy Bucharest, 37 Dionisie Lupu, 020021 Bucharest, Romania; r.bohiltea@yahoo.com (R.B.); or nicolaebacalbasa@gmail.com (N.B.); valentin.varlas@umfcd.ro (V.V.); 2 Department of Obstetrics and Gynecology, University Emergency Hospital Bucharest, 169 Splaiul Independentei Bld., Sector 5, 050098 Bucharest, Romania; 3 Department of Obstetrics and Gynecology, Filantropia Hospital, 11-13 Ion Mihalache Blv., Sector 1, 011171 Bucharest, Romania; bmmihai@gmail.com; 4 Optospintronics Department, National Institute for Research and Development in Optoelectronics-INOE 2000, 409 Atomistilor, 077125 Magurele, Romania; stefan.iordache@inoe.ro (S.-M.I.); krisis812@yahoo.co.uk (C.E.A.G.); 5 Department of Interventional Radiology, University Emergency Hospital Bucharest, 169 Splaiul Independentei Bld., Sector 5, 050098 Bucharest, Romania; bdorobat@gmail.com; 6 Department of Interventional Radiology, Life Memorial Hospital, 050098 Bucharest , Romania; 7 Faculty of Medicine, “Carol Davila” University of Medicine and Pharmacy Bucharest, 37 Dionisie Lupu, 020021 Bucharest, Romania; vladareanu@gmail.com; 8 École Hôtelière de Lausanne, 1000 Lausanne, Switzerland; alexia.bohiltea@ehl.ch

**Keywords:** cesarean scar pregnancy, invasive placenta, uterine artery embolization, CSP registry

## Abstract

Objective: The aim of this study is to propose a standardized management of care for patients diagnosed with cesarean scar pregnancy (CSP). There are two types of CSP: Type 1 (on the scar) vs. type 2 (in the niche). To date there is no international standard to predict the extent of invasion or the optimal management of CSP. Materials and methods: We used intramuscular methotrexate injection followed by uterine artery embolization combined with suction evacuation as a conservative approach for the treatment of seven patients diagnosed with CSP. Our inclusion criteria, to be satisfied simultaneously, were established as follows: (1) patients with CSP; (2) early gestational age ≤ 9 weeks, and (3) written consent of the proposed treatment of the patient. Results: This course of treatment produced a positive outcome in all cases. We did not have any complications (e.g., emergency hysterectomy, perforation of the uterine cavity, severe hemorrhage, or endometritis) during the procedures or in the follow-up. The most important predictors of successful management are early diagnosis of CSP and orientation of the invasive trophoblast opposite to the scar. Conclusions: The main finding from this series of cases is that associating systemic methotrexate and uterine artery embolization provides efficient and low-risk management of CSP. This treatment regime is adequate for both types of CSPs. We consider that early localization diagnosis of pregnancy following a cesarean delivery is mandatory for CSP morbidity prevention.

## 1. Introduction

According to the Society for Maternal–Fetal Medicine, cesarean scar pregnancy or CSP is a severe complication, in which “early pregnancy is grafted in the scar from a prior cesarean delivery” [1]. Recognized as true ectopic pregnancy even if the gestational sac is developing in the uterine cavity, the CSP carries major maternal morbidity and the optimal treatment is almost always pregnancy termination. The area around the cesarean scars has a reduced thickness of the myometrium (usually below 2 mm), which allows the trophoblastic cells to migrate near the uterine serosa and remodel the spiral and radial arteries in the deep myometrium [2]. This produces a hypervascularization at a subplacental level and leads to an increased risk of uterine rupture and accreta spectrum placentation. Around 6–10 weeks of gestation age, the size of the ectopic implanted gestational sac starts deviating from normality, followed by changes in the fetal heart rate and crown-rump length (CRL) [3]. 

Our group participates as a member in the Cesarean Scar Pregnancy (CSP) registry conducted by St. George’s University of London together with other healthcare centers worldwide. The international CSP registry is oriented towards women who received care at obstetrics/gynecologic (OB/GYN) hospitals; patients diagnosed with CSP by ultrasound examination; and patients who have completed treatment and management of CSP, either delivered by cesarean section or terminated their pregnancy. The diagnosis criteria used for the inclusion are laid down as follows: (1) positive pregnancy test; (2) visualization of an empty uterine cavity as well as an empty endocervical canal on transvaginal ultrasound; (3) detection of the placenta and/or a gestational sac embedded in the hysterotomy scar; (4) embryonic/fetal pole and/or yolk sac (+/− heart activity) in the gestational sac, seen on transvaginal ultrasound; (5) triangular gestational sac filling the niche of the scar before 8 weeks’ gestation; after 8 weeks’ gestation this shape becomes more rounded and part or the entire chorionic sac can be seen approaching into the uterine cavity, the placenta and its blood flow remaining within or on the scar and defining the positive diagnosis of CSP; (6) a thin (1–3 mm) or absent myometrial layer between the gestational sac and the bladder seen on transvaginal ultrasound defined as residual myometrium thickness [4].

There are two types of CSP. The diagnosis of CSP Type 1 (on the scar) vs. type 2 (in the niche) is defined if >50% of the gestational sac protrudes towards the uterine cavity/cervical canal, whereas type 2 CSP is defined by the trophoblastic implantation into a deficient or dehiscent scar and the protrusion of the gestational sac is ≤50% [5].

The manner in which the termination is performed has a direct impact on the future of the reproductive capacity. The recommendations from the Society for Maternal–Fetal Medicine suggest two types of management for this situation: surgical treatment (resection) or medical (Methotrexate) followed by surgical treatment [1,6]. Curettage alone or systemic methotrexate administration, as well as expectant management, are strongly considered unsuitable because of the high morbidity and mortality maternal risks (hemorrhage, perforation, development of uterine arteriovenous malformation, etc. [7,8,9,10,11]). However, there is no consensus for the optimal treatment regime, and different situations require different approaches [12,13]. Recently, Tam Tam et al. proposed a multidisciplinary approach based on the administration of methotrexate, using a computed tomography-guided injection [14], which showed promising results in term of minimal invasion procedure and low risk associated with pregnancy termination. Yuan et al. [15] proposed a focused ultrasound ablation method combined with ultrasound-guided suction curettage for the treatment of CSP. Other methods include laparoscopic management [16,17], sometimes with robotic assistance [18,19], ligation/clamping of uterine arteries [20], lesion resection [21], and transvaginal hysterotomy [22]. Some protocols propose vacuum aspiration under ultrasound guidance [23,24], due to the low blood loss while other authors recommend administration of multidose regimen of methotrexate [25,26,27] combined with curettage [28]. 

As one can see, there is not a consensus in the management of the CSP, each clinic and hospital has its own protocol. A recent study analyzing the clinical efficacy of different therapeutic methods for the treatment of CSP indicated that uterine artery embolization has the highest success rates, with minimal intraoperative blood loss [29]. Blood loss is an important risk that needs to be addressed since it can lead to an emergency hysterectomy and the loss of fertility. Generally speaking, hysterectomy is performed when massive hemorrhage occurs. Uncontrollable bleeding may be the result of either a major complication during surgical treatment of CSP, or a result of uterine rupture caused by CSP. Since the fertility is completely lost, this procedure is reserved only for the most dire cases. According to Timor-Tritsch et al. [5] this happens in 3.7% of CSP cases diagnosed before 9 weeks of pregnancy and 16.3% of cases diagnosed in the late first trimester. 

We focused on seven cases that have shown themselves in the OB/GYN clinic for consultation and were identified as CSP. Our proposed management approach is based on uterine artery embolization after methotrexate administration and followed by vacuum aspiration. Table 1 presents the course treatment and prognosis for the seven cases.

## 2. Materials and Methods

The study was conducted at the University Emergency Hospital in Bucharest for a time period of two years (from 2020 and 2021) and was approved by the Ethics Committee of University Emergency Hospital (protocol code 26619/04.06.2020).

**Figure 1:**
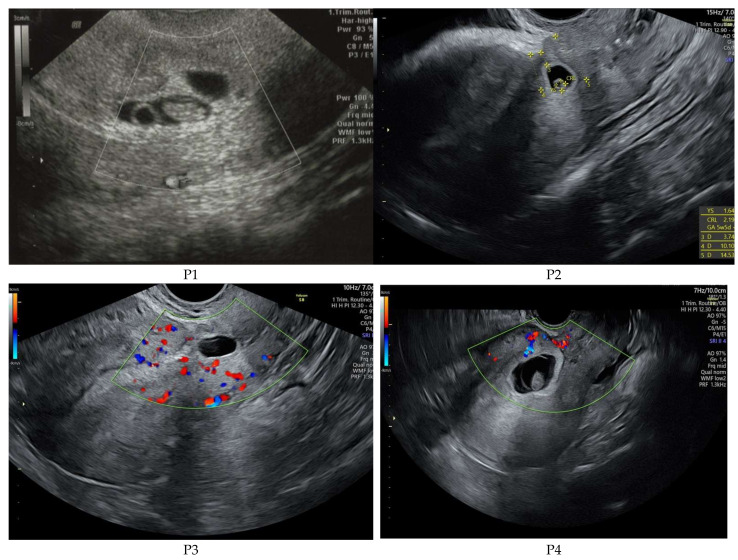

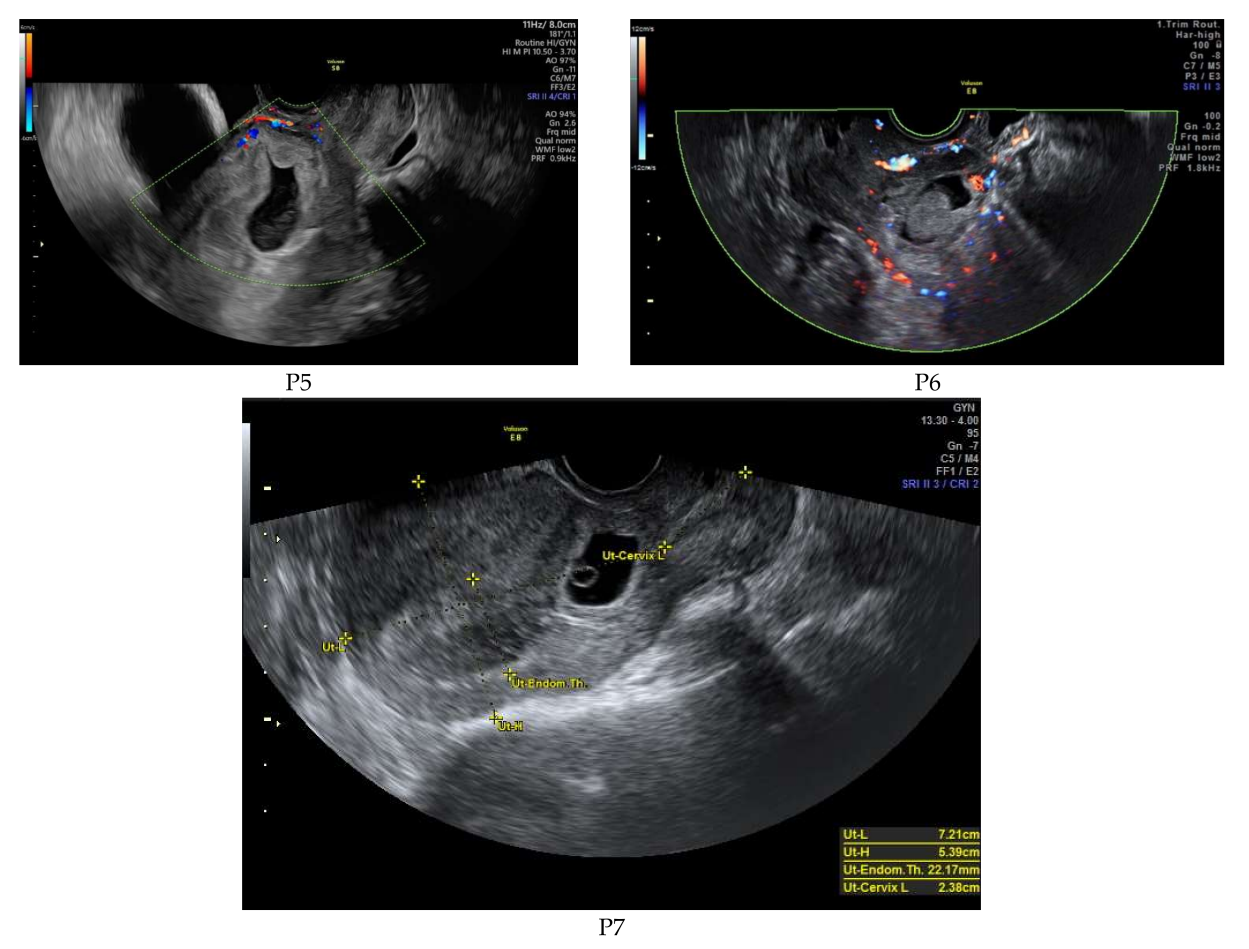
CSP ultrasound imaging for the cases presented in this study before treatment.

Per standard of care in our group, patients suspected of CSP first undergo 2D transabdominal or transvaginal gray scale and color Doppler ultrasound; investigation is performed with a Voluson E8 (GE Medical Systems Kretztechnik GmbH & Co Ohg, Tiefenbach, Austria), equipped with a Ge Rab4-8-D Ultrasound Probe (Figure 1). Our national protocol supports the early diagnosis of pregnancy, managed by ultrasound evaluation for viability and localization of gestational sac; in the cases of vaginal bleeding associated with early pregnancy, differential diagnosis is achieved by using color Doppler ultrasongraphy. The second stage is the evaluation of the β-hCG (Human Chorionic Gonadotropin) serum value. According to the ultrasound results, and subsequent personal, evidence-based counseling session, the patient’s goals for pregnancy management are outlined (i.e., conservative follow-up of continued pregnancy, or termination). We administrate 1 mg of Methotrexate/kg body weight by intramuscular route to cease trophoblastic proliferation, followed by transvaginal ultrasound examination and uterine artery embolization in the interventional radiology department, taking into account the patient’s renal function and possible allergy to iodine. After achieving the uterine arteries occlusion, we repeat transvaginal ultrasound Doppler evaluation of both trophoblastic invasion of the uterine wall defect and uterine arteries flow, and within the next 48 h, we perform ultrasound guided vacuum suction of the uterine cavity. Dynamics of β-hCG is monitored until non-pregnancy values are reached.

**Figure 2:**
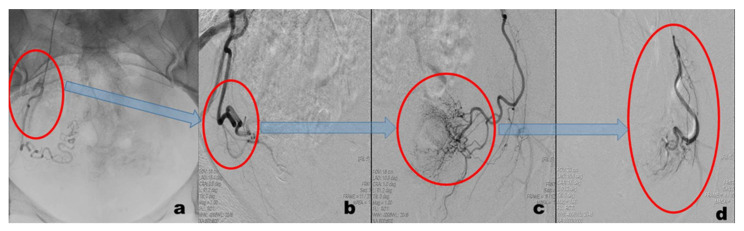
Embolization of the uterine arteries following a few simple steps, defined in the red circle: (**a**) introduction of a 5F Roberts catheter in the right femoral artery; (**b**) X-ray guidance of the catheter to the point of embolization; (**c**) release of polyvinyl alcohol or gelfoam particles to block the blood circulation in the placenta; and (**d**) blockage of the circulation system in the developing placenta.

Embolization of uterine arteries (Figure 2, performed with LP Angio Digital from GE Medical Systems S.C.S., Buc, France) is achieved in less than one hour by following a few simple steps: (a) local anesthesia around the right femoral artery; (b) introduction of a 5F Roberts catheter in the right femoral artery; (c) X-ray guidance of the catheter to the point of embolization; (d) polyvinyl alcohol or gelfoam particles mixed with contrast are injected through the catheter and flow directed to block the blood circulation in the placenta; and (e) retraction of the catheter and wound dressing. Embolization is considered complete when the flow is arrested. The intermittent pain associated with UAE, and described by the patient as mild to severe, appears immediately after embolization and intensified in the first 6 h, gradually decreasing in the next 24–48 h; it is often associated with nausea and vomiting. Management of pain and other symptoms required the administration of anti-inflammatory drugs, analgesics, intravenous opioids, and antiemetics. 

For this study, our inclusion criteria were established as follows: (1) patients with CSP, (2) early gestational age ≤ 9 weeks, and (3) written consent of the proposed treatment of the patient. None of the patients refused the proposed procedure once they understood the risks of continuing the pregnancy when compared to the risk of bleeding following curettage, or compared to the invasiveness of alternative surgical procedures.

## 3. Results

The treatment used was intramuscular methotrexate injection followed by uterine artery embolization as a first line method combined with suction evacuation of the uterine cavity. The incidence of CSP was calculated at 2 CSP pregnancies at every 10,000 pregnancies (we had 7 cases at a mean 18,000 pregnancies per year for our hospital). Two of the seven cases of CSP were diagnosed during the first pregnancy evaluation, three CSP were referred from other hospitals to our center for the benefit of the interventional radiology care unit, and two came in the clinic with vaginal bleeding. Two of the patients had developed CSP after a single cesarean delivery (CD), four—after two CDs and one patient had had three previous CDs (C-sections). For all the patients the previous C-sections were single-layer closure. The usual treatment used, as we described above was intramuscular methotrexate injection followed by uterine artery embolization combined with suction evacuation as surgical approach. 

Considering the financial aspect of the proposed approach, we looked at the estimative costs in Romania, West Europe, and USA. The uterine artery embolization technique has an estimative cost of EUR 677.37 in Romania, which include the costs for: (a) one day inpatient treatment (hospitalization); (b) the use of one dose of methotrexate; (c) the interventional radiology; and (d) vacuum aspiration.

The diagnostic of the CSP was performed by ultrasound means. Following the results obtained by Timor-Tritsch et al. [5] we measured the residual myometrial layer between the gestational sac and bladder and established the presence or absence of fetal heartbeat as well as the presence/absence of rich vascular pattern in the placenta. Our results are presented in Table 2. It should be noticed that for P4 we decided against uterine artery embolization (UAE) due to low vascularization of the scar site determined by low gestational age and Type 1 CSP.

This course of treatment produced a positive outcome in all cases. We did not have any complications (e.g., emergency hysterectomy, perforation of the uterine cavity, severe hemorrhage, or endometritis) during the procedures or in the follow-up. The average time (in days) from peak β-hCG to 0 was 40.5 with a maximum and minimum interval of 50 to 30 days.

## 4. Discussion

Diagnosis of the CSP is performed by ultrasound means, following well-established criteria [30,31]; however, there is not a standardized protocol for the treatment of CSP. Some authors associated uterine artery embolization (UAE) with dilatation and curettage [32,33,34,35,36], and the procedure was uneventful in most of the cases, with minimal blood loss (≈23 mL). Curettage could be the common intervention but patients have a high risk of developing massive hemorrhage, which might lead to open surgery. Zhang et al. [32] studied 15 patients diagnosed with CSP who were treated with UAE and curettage. Their results showed that UAE is an efficient method for controlling the bleeding and for causing ischemic death of CSP and should be performed as first choice of treatment in emergency care. However, the administration of methotrexate alone and/or combined with curettage causes uncontrollable bleeding (4 cases out of 15 were misdiagnosed and treated by bling curettage, which induced heavy bleeding and required emergency UAE). This is supported by Lou et al. [35], who studied 53 women treated with methotrexate and UAE, followed by curettage. Their results showed that the blood volume lost during curettage was 23–61 mL and hemorrhage occurred in two women. Their conclusion was that although curettage is the most commonly used treatment method for CSP, direct curettage without optimal pretreatment can lead to adverse clinical outcomes such as heavy bleeding. This is sustained by several studies that highlight the increased risk of severe vaginal bleeding (with subsequent need for hysterectomy) [37,38].

The reason behind our guided approach (MTX→UAE *vs* UAE→MTX) takes into account that the distribution of methotrexate among trophoblastic cells is diminished if it follows UAE. This allows a low degree of personalization of treatment and its tailoring to the needs of the patient. Three important studies [33,34,36] showed that UAE combined with methotrexate has superior outcomes compared to surgery in terms of blood loss and success rate, as well as hospital stay. In terms of surgery time, surgical management of CSP (this includes suction and curettage) were similar with UAE, and established at 29 min. 

Suction evacuation, as a second line of treatment, takes place with minimum blood loss, well below the level of bleeding resulted from other treatment routes (300 to 1200 mL) [39]. Following our proposed treatment line, all our patients were released with the same level of hemoglobin as during admission. 

**Figure 3:**
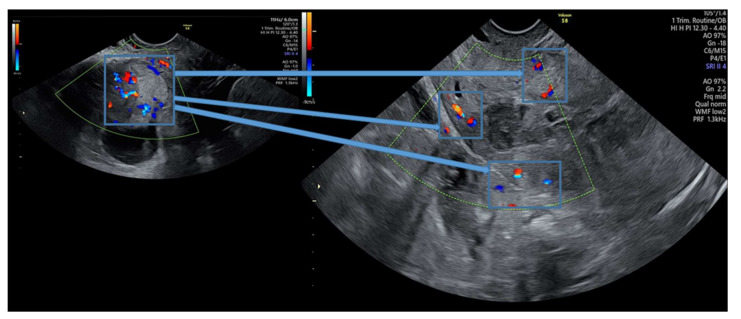
Doppler ultrasound before and after two steeps procedure showing the minimum residual vascularization (the areas of interest are shown post-procedure highlighted in the blue squares connected to the initial area via blue arrows).

**Figure 4:**
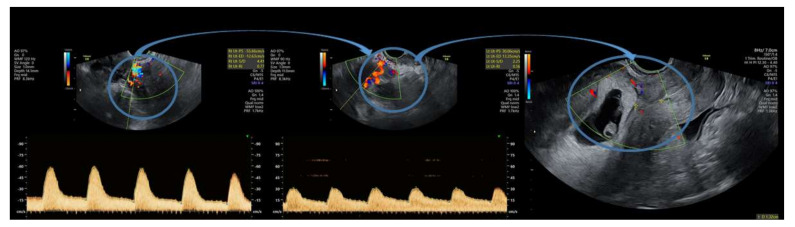
Progression of circulation after successful uterine artery embolization for P6 (shown inside the blue circles, connected via arrows). Complete cession of blood flow occurred after 48 h (artery flow is restored after 24 h since embolization, but then is completely reduced at 48 h).

An interesting observed fact is that uterine artery flow at 24 h post-embolization shows normal velocity parameters; however, even a small temporary restriction of blood circulation in the uterine arteries has proven efficient in reducing trophoblastic cell vascularization and abruptly decreasing the levels of β-hCG (Figure 3). During radiologic procedure, the post-embolization vascular obstruction is always tested and documented (Figure 4). 

The outcome for our approach showed no complications during treatment or afterwards in the follow-up. The patients are discharged 24 h after the minimum-invasive treatment. In two of our cases, (P1 and P7) obtained a subsequent normal pregnancy resulted in a term live birth, one year after successful treatment of CSP. 

### 4.1. Financial Aspects

In Western Europe and United States, the costs for the same procedure are on an average EUR 5130. For Romania and Western Europe those costs are mainly sustained through the National Health System (NHS), whereas in USA those costs are supported by the patients themselves or by their insurance companies. Comparing these estimative costs to the costs of an emergency hysterectomy (totaling EUR 1830 in Romania without ICU care, and around EUR 6620 for the US and Western Europe including three days of hospitalization and surgical procedure), one can easily observe that the cost of our proposed treatment is significantly lower than that of the treatment and complications associated with D&C/expectative management. According to the studies discussing curettage, the risk of bleeding associated with this procedure is major, and leads in the majority of cases to the implementation of a surgical therapeutic procedure, whose costs are those related to hysterectomy/excision intervention. The excisional intervention in Romania is coded for a duration similar to a hysterectomy, implying similar costs. Expectant management can be applied to CSP, which has spontaneously stopped evolving, in which case the costs are related to the serial repetition of β-hCG for a period of minimum 30 days until negativity. Expectant management at the request of the patient in the case of continuation of pregnancy, involves premature birth and neonatal prematurity, whose costs vary between EUR 10 and 40k (these costs include the costs of hysterectomy and intensive care). These are estimative costs and do not include psychological impact inducted by the loss of fertility and blood transfusions that may be needed during the emergency intervention procedures.

### 4.2. Study Strengths

The strengths of this study refer to the combination of proposed techniques, which, brought together, offer a high successful rate. The methodology is minimally invasive, associated with minimal bleeding, has lower costs, and allows complete acceptability for patients (three of the cases presented were referred from other centers for the availability of our interventional radiology service). 

### 4.3. Study Limitations

The limitations of this study are: (a) the low number of enrolled patients (because CSP is a rare type of ectopic pregnancy, and because we achieved the proposed protocol after many others in the previous years, including MTX associated with curettage, UEA followed by expectant management) → a mitigation action will be the inclusion of more women over a larger period of time; and (b) the proposed treatment course is a multidisciplinary action that involves collaboration between departments of OB/GYN and interventional radiology, where the latter is not available in many hospitals.

## 5. Conclusions

The main finding of this series of cases is that association of systemic methotrexate, uterine artery embolization, and suction evacuation gives efficient and low-risk management of CSP. This treatment regime is adequate for both types of CSP (type 1 and type 2). No complications resulted from the treatment. It is worth noting that this management route represents a conservative solution for the patients who desire conservation of fertility, provided that the correct diagnosis is established. The preservation of fertility through the use of embolization is sustained by the evolution of P1 and P7. Doppler transvaginal ultrasound of CSP in early pregnancy certifies the presence of ectopic invasion of the trophoblast in the uterine scar, thus disproving other differential diagnosis, such as ongoing abortion. This correct assessment of the diagnosis avoids complicated abrasive curettage, which can lead to massive hemorrhage. The most important predictors of successful management are early diagnosis of CSP and orientation of the invasive trophoblast opposite to the scar. We consider that early localization diagnosis of pregnancy following a cesarean delivery is mandatory for CSP morbidity prevention.

## Figures and Tables

**Table 1:** Anonymized patient data.

Patient No	Peak bHCG Value	Gestational Age	CSP	Treatment Course	Outcome
P1 *	99,999	7w + 1d	Type 1	1. Intramuscular methotrexate injection 2. Uterine artery embolization 3. Suction evacuation	No complications
P2	99,999	5w + 2d	Type 2	1. Intramuscular methotrexate injection 2. Uterine artery embolization 3. Suction evacuation	No complications
P3	62,309	7w + 2d	Type 2	1. Intramuscular methotrexate injection 2. Uterine artery embolization 3. Suction evacuation	No complications
P4	5938	5w + 5d	Type 1	1.Intramuscular methotrexate injection 2. Suction evacuation	No complications
P5	4579	5w + 6d	Type 2	1.Intramuscular methotrexate injection 2. Uterine artery embolization 3.Suction evacuation	No complications
P6	70,373	6w + 5d	Type 1	1. Intramuscular methotrexate injection 2. Uterine artery embolization 3. Suction evacuation	No complications
P7	46,317	6w + 2d	Type 2	1. Intramuscular methotrexate injection 2. Uterine artery embolization 3. Suction evacuation	No complications

* Among the 7, two of CSP were diagnosed during the first pregnancy evaluation, three CSP were referred from other hospitals, and 2 patients presented in the clinic with vaginal bleeding. P1 had a twin pregnancy with both embryos in the scar. Only P3 and P7 had a fetal heartbeat present and was diagnosed with placenta lacunae.

**Table 2:** The results of the initial ultrasound evaluation.

Patient No	Residual Myometrium Thickness (mm)	Fetal Heartbeat	Vascularization	Crown-Rump Length (mm)	Gestational Sac Diameter (mm)	BMI
P1 *	2	absent	increased	11	22/20	27.5
P2	2.1	absent	increased	Not measured	11	22.5
P3	1.6	present	increased	13	24	28.4
P4	3.3	absent	normal	2	8	33.6
P5	4	absent	normal	Not measured	8	29.4
P6	3.8	absent	increased	4	23	19.7
P7	3.5	present	increased	5	28	21

* P1 presents two values for the gestational sac diameter (one for each of the twin embryos).
